# Co-localization of Carbonic Anhydrase and Phospho*enol*-pyruvate Carboxylase and Localization of Pyruvate Kinase in Roots and Hypocotyls of Etiolated *Glycine max* Seedlings

**DOI:** 10.3390/ijms10072896

**Published:** 2009-06-29

**Authors:** Maria Dimou, Anca Paunescu, Georgios Aivalakis, Emmanouil Flemetakis, Panagiotis Katinakis

**Affiliations:** 1 Department of Agricultural Biotechnology, Agricultural University of Athens, Iera Odos 75, 11855 Botanikos, Athens, Greece; E-Mails: mdimougr@yahoo.com (M.D.); gaivalakis@aua.gr (G.A.); mflem@aua.gr (E.F.); 2 Institute of Biology, Splaiul Independentei 296, Bucharest 060031, Romania; E-Mail: ancuta_paun@yahoo.com (A.P.)

**Keywords:** carbonic anhydrase, Glycine max, hypocotyl, *in situ* hybridization, phosphoenolpyruvate carboxylase, pyruvate kinase, root

## Abstract

We investigated the presence of carbonic anhydrase in root and hypocotyl of etiolated soybean using enzymatic, histochemical, immunohistochemical and *in situ* hybridization approaches. In parallel, we used *in situ* hybridization and immunolocalization to determine the expression pattern and localization of phospho*enol*pyruvate carboxylase. Their co-localization in the root tip as well as in the central cylinder, suggests that a large fraction of the CO_2_ may be re-introduced into C4 compounds. *GmPK3* expression, coding for a cytoplasmic isoform of pyruvate kinase, was detected in all different root cell types, suggesting that both phospho*enol*pyruvate-utilizing enzymes are involved in phospho*enol*pyruvate metabolism in etiolated soybean roots; a case indicative of the necessary flexibility plant metabolism has to adopt in order to compensate various physiological conditions.

## 1. Introduction

Carbonic anhydrase (CA; EC: 4.2.1.1) is a zinc-containing enzyme that catalyzes the reversible of CO_2_ to HCO^−^_3_ and has been found in the three domains of life: archaea, bacteria and eukarya. So far, four distinct CA classes have been reported namely α-, β-, γ- and δ-CA [1,2]. A fifth class, named ɛ-CA, is found in several marine cyanobacteria and chemolithoautotrophic bacteria [3] but recent studies indicated that this class is a variant of β-CA [4]. In plants, all known CAs belong to classes α, β and γ, with the β class being the predominant [5–7].

In photosynthetic organisms, the carbonic anhydrases are involved in diverse physiological processes such as ion exchange, acid/base balance, carboxylation/decarboxylation reactions and inorganic carbon diffusion between the cell and its environment as well as within the cell [8]. In C3 plants, most CA activity is localized to the chloroplast stroma of the mesophyll cells and may be responsible for maintaining adequate concentrations of CO_2_ around ribulose-bisphosphate carboxylase (Rubisco, EC 4.1.1.39) by facilitating the diffusion of CO_2_ across the chloroplast envelope or by rapidly dehydrating bicarbonate to CO_2_ [9,10]. In contrast, a cytoplasmic β-CA facilitates the hydration of atmospheric CO_2_ to bicarbonate, the substrate of phospho*enol*pyruvate carboxylase (PEPC; EC 4.1.1.31), the primary carboxylating enzyme for C4 or CAM photosynthesis [11–13].

PEPC catalyzes the β-carboxylation of PEP, in the presence of Mg^2+^, to yield oxaloacetate and inorganic phosphate [12]. The active enzyme acts as a homotetramer, of approximately 400 kDa, in the cytoplasm of plant cells and is encoded by small gene families whose members show differential expression in plant organs [14]. C4 and CAM plants, apply PEPC for the initial fixation of atmospheric CO_2_, while in leaves of C3 plants; the enzyme is involved in the anaplerotic replenishment of tricarboxylic acid cycle intermediates [15]. Furthermore, PEPC isoforms, also known as C3 or root isoforms are highly expressed in non-green tissues of C3 plants such as roots and legume root nodules [16–19]. These isoforms evolved from the anaplerotic (C3) enzyme, but show different expression levels and tissue specificities. Also, they differ in their catalytic properties concerning substrate affinity and allosteric regulation by metabolic intermediates [20].

In such nonphotosynthetic tissues, respiration is considered an important source of CO_2_ to supply the PEPC catalyzed reaction. Nevertheless, if the uncatalyzed rate of CO_2_ conversion to HCO^−^_3_ is not sufficiently high, enzymatic hydration of CO_2_ to HCO^−^_3_ by carbonic anhydrases is necessary [21]. A temporal and spatial association of cytoplasmic β-CA with PEPC expression supports their functional coupling into refixing the respired CO_2_ in highly respiring non-green tissues, such as legume seeds and young nodules [15,16,22,23].

Even though, PEPC has been considered as the main plant anaplerotic enzyme [24], pyruvate kinase (PK, EC 2.7.1.40) can, as well, utilize PEP converting it to pyruvate, which is a key metabolic intermediate of many pathways, such as energy production and the biosynthesis of amino acids, organic acids and fatty acids [25]. PK exists as cytosolic and plastidic isoforms with diverse physical, immunological and kinetic characteristics [25]. Tissue-specific cytosolic PK isoforms have been demonstrated considerable differences in their kinetic and regulatory properties reflecting the particular metabolic requirements of the respective tissues [26].

Roots and hypocotyls of etiolated seedlings are actively growing and highly respiring heterotrophic tissues where the possible association of CA with PEPC could be further studied. Although it has been considered that a PEPC mediated CO_2_ fixation is taking place in C3 plant roots [18,27,28]; and the presence of CA has been confirmed there [7,29,30], even if in some cases CA activity could not be detected [31], the precise cellular localization of both enzymes still has not been adequately investigated. In the present study, the presence of CA in roots and hypocotyls of etiolated soybean seedlings was examined using enzymatic, histochemical, immunohistochemical and *in situ* hybridization approaches, while the cellular localization of PEPC was investigated using both immunohistochemical and *in situ* hybridization approaches. In addition, the temporal and spatial gene expression patterns of pyruvate kinase (*GmPK3*) were investigated as the enzyme utilizes the same substrate with PEPC.

## 2. Results

### 2.1. CA activity and histochemical localization of CA activity in etiolated G. max seedlings

The presence of CA enzyme activity, in soybean roots and etiolated hypocotyls, was investigated spectrophotometrically. The data indicated that CA activity was observed in most tissues examined, with significantly higher levels detected in roots of etiolated soybean plants as compared to that observed in hypocotyls (p<0.05, Table 1).

**Figure 1.**
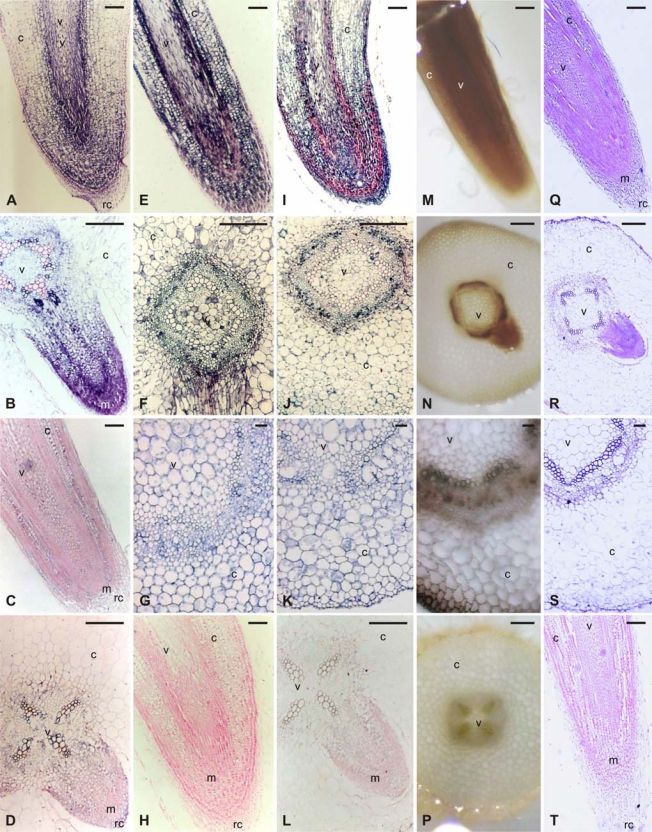
*In situ* localization of *GmPK3*, *GmPEPC7* and *GmCA1* gene transcripts, immunohistochemical localization of PEPC and CA proteins and histochemical localization of total CA activity in primary and lateral roots of 8-days old etiolated soybean seedlings. *In situ* hybridization and immunohistochemical signals are visible as blue-purple precipitate. Histochemical localization signal is observed as brown-black signal. (A) Longitudinal section from the root tip region hybridized with the antisense probe for the *GmPEPC7* gene. (B) Cross section of the primary root bearing the lateral root primordia (approximately 4.5 cm from the primary root tip) hybridized with the antisense probe for the *GmPEPC7* gene. (C) Longitudinal section from the root tip region hybridized with the antisense probe for the *GmPK3* gene. (D) Cross section of the primary root bearing the lateral root primordia (approximately 4.5 cm from the primary root tip) hybridized with the antisense probe for the *GmPK3* gene. (H) Longitudinal section from the root tip region hybridized with the sense probe for the *GmPK3* gene. (Q) Longitudinal section from the root tip region hybridized with the antisense probe for the *GmCA1* gene. (R) Cross section of the primary root bearing the lateral root primordia (approximately 4.5 cm from the primary root tip) hybridized with the antisense probe for the *GmCA1* gene. (S) Cross section from the hypocotyls hybridized with the antisense probe for the *GmCA1* gene. (T) Longitudinal section from the root tip region hybridized with the sense probe for the *GmCA1* gene. (E) Immunohistochemical localization of PEPC protein using polyclonal antibodies raised against GmPEPC7 in longitudinal section from the root tip region. (F) Immunohistochemical localization of PEPC protein using polyclonal antibodies raised against *Gm*PEPC7 in cross section of the primary root bearing the lateral root primordia (approximately 4.5cm from the primary root tip). (G) Immunohistochemical localization of PEPC protein using polyclonal antibodies raised against *Gm*PEPC7 in cross section from the hypocotyl. (I) Immunohistochemical localization of CA protein using polyclonal antibodies raised against *Gm*CA1 in longitudinal section from the root tip region. (J) Immunohistochemical localization of CA protein using polyclonal antibodies raised against *Gm*CA1 in cross section of the primary root bearing the lateral root primordia (approximately 4.5 cm from the primary root tip). (K) Immunohistochemical localization of CA protein using polyclonal antibodies raised against *Gm*CA1 in cross section from the hypocotyl. (L) In immunolocalization control reaction cross section of the primary root bearing the lateral root primordia (approximately 4.5 cm from the primary root tip) was incubated with pre-immune serum. (M) Histochemical localization of CA activity in longitudinal section from the root tip region. (N) Histochemical localization of CA activity in cross section of the primary root bearing the lateral root primordia (approximately 4.5 cm from the primary root tip). (O) Histochemical localization of CA activity in cross section from the hypocotyl. (P) In histochemical assay control reaction cross section of the primary root (approximately 4.5 cm from the primary root tip) was incubated with the reaction mix in the absence of NaHCO_3_. rc, root cap; v, vascular cylinder; m, meristem; c, cortex. Scale bars: 200 μm.

The spatial distribution of CA activity was visualized by an *in situ* staining for activity in fresh sections of *G. max* roots and hypocotyls. Activity of the enzyme is coupled to the formation of a black precipitate. High levels of CA activity were detected at the meristematic zone of the primary and lateral root (Figures 1M and N). Less staining was detected in metaxylem vessels but also in phloem and in the middle enlarging cortical cells for both root and hypocotyls (Figures 1N and O). As a negative control, the histochemical assay was performed in the absence of NaHCO_3_ (Figure 1P).

### 2.2. Immunohistochemical detection of CA and PEPC protein

**Figure 2.**
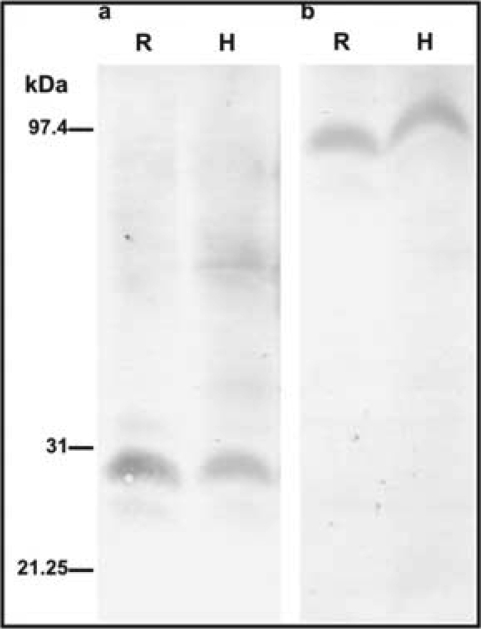
Western blots of *G. max* CA and PEPC proteins. *G. max* root (R) and hypocotyls (H) proteins were separated by 15% SDS-PAGE, transferred to nitrocellulose, and probed with polyclonal antibodies raised against *Gm*CA1 coding for a beta-type cytoplasmic CA (a) and *Gm*PEPC7 coding for a cytoplasmic PEPC (b). Molecular mass markers are shown in kDa.

Extracts from roots and hypocotyls of etiolated seedlings were analyzed by Western blot using the polyclonal antibodies raised against *Gm*CA1 coding for a beta-type cytoplasmic CA and *Gm*PEPC7 coding for a cytoplasmic PEPC [16]. Both antibodies detected a polypteptide of about 29 and 100kDa respectively, in both roots and hypocotyls (Figure 2). Using the polyclonal antibody raised against *Gm*CA1, CA was previously localized in the cytoplasm of the inner cortical cells and the cell walls of the endodermal cells of mature soybean nodules [32].

The spatial distribution of CA and PEPC protein in the different cell types of roots and hypocotyls of etiolated seedlings was examined by immunohistochemical localization. Transverse and longitudinal root sections as well as transverse hypocotyls sections were incubated with polyclonal antibodies raised against *Gm*CA1 and *Gm*PEPC7. It was observed that the PEPC protein was detected in all tissues. However, the signal was higher in cells of the region of cell division, but in the region of elongation it was restricted at the vascular cylinder and in a few neighboring cell layers (Figure 1E). The localization of CA protein essentially followed the patterns of localization of PEPC protein, with higher expression in developing metaxylem vessels, sieve elements and enlarging cortical cells, for both root and hypocotyls (Figure 1J and K). Furthermore, high signal was detected at the root apical meristem (Figure 2I). When sections were incubated with pre-immune serum, as a negative control, no significant signal was detected (Figure 1L).

### 2.3. Spatial patterns of GmCA1, GmPEPC7 and GmPK3 transcripts in primary and lateral roots of etiolated G. max seedlings

**Figure 3.**
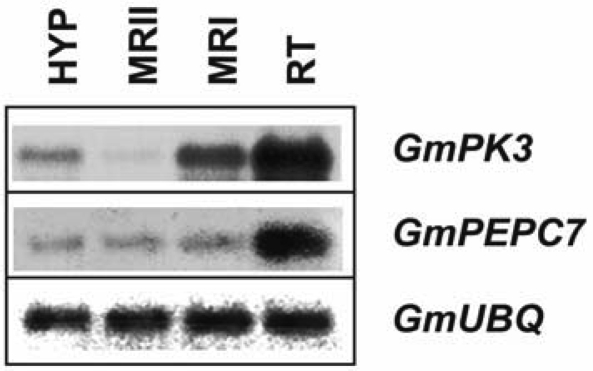
Semi-quantitative reverse transcription polymerase chain reaction analysis of the expression profiles of *GmPK3* and *GmPEPC7* genes. Total RNA from hypocotyls (HYP), and different parts of roots, representing the region of cell division (RT), the root region of elongation (MRI) and the root region containing the lateral root primordia (MRII) of 8-days old etiolated seedlings was subjected to reverse transcription polymerase chain reaction analysis. The RT-PCR product of the *GmUBQ* was used as an internal control. Amplified products were separated on 1.5% agarose gels, blotted on nylon membranes and hybridized with the respective digoxigenin-11-rUTP labeled cDNA probes.

To confirm the presence of *GmPEPC7* and *GmPK3* gene transcripts in roots (the root region of cell division, elongation, and the root region of laterals emergence) and hypocotyls of 8-days old etiolated soybean seedlings, we used a semi quantitative reverse transcription-PCR approach. A housekeeping gene coding for ubiquitin was used as an internal control. As shown in Figure 3, *GmPEPC7* gene transcripts were detected in all parts of the root examined and hypocotyls, with the highest levels of accumulation to be detected in the root tip region of 8-day old etiolated seedlings. *GmPK3* transcript accumulation was observed in all tissues tested with the highest levels in the root tip region (RT) of 8-day old etiolated seedlings while very low levels compared to the other tissues, were detected in the root region of laterals emergence (MRII).

Having established the presence of *GmPK3* and *GmPEPC7* gene transcripts, their spatial localization during root development was investigated using an *in situ* hybridization method. Longitudinal sections of primary and lateral roots of 8-day old etiolated soybean seedlings were hybridized with 11-digoxigenin-rUTP-labeled RNA probes transcribed from *GmPK3* and *GmPEPC7* cDNA clones. *GmPK3* (Figure 1C and D) and *GmPEPC7* (Figures 1A and B) expression was detected in the root tip region and mostly in the developing cortex parenchyma of root tip and in the central root cap region of the primary and lateral roots. Levels of both transcripts, in the vascular tissue and in a few cell layers neighbouring the vascular tissue, gradually increased from the region of cell division to the region of elongation and then decreased in the region of laterals emergence. *GmPK3* and *GmPEPC7* transcripts were detected in most cells in the region of cell division of lateral roots, but as the cells differentiated; *GmPK3* and *GmPEPC7* expression was confined to the vascular cylinder (Figures 1B and D). Moreover, the signal for both gene transcripts was very high in developing xylem vessels. As a negative control, sections were hybridized to sense RNA probes transcribed from the corresponding cDNAs. In that case, no significant signal was detected (Figure 1H).

Subsequently, the *GmCA1* gene expression pattern was studied using the *in situ* hybridization method. Essentially, *GmCA1* transcripts localization followed the patterns of localization of CA protein and activity, with higher expression in developing metaxylem vessels, sieve elements and enlarging cortical cells, for both root and hypocotyls (Figures 1R and S). Furthermore, high signal was detected at the root apical meristem (Figure 1Q) the root epidermis and some underlying cell layers (Figures 1R and S). When sections were incubated with sense probe for the *GmCA1* gene, as a negative control, no significant signal was detected (Figure 1T).

## 3. Discussion

Although it has been suggested that the uncatalyzed hydration of CO_2_ occurs at rates sufficient to support the activities of root PEPC [21], inhibitors of CA activity inhibited respiratory O_2_ consumption and DI^14^C (DIC: dissolved inorganic carbon, CO_2_+HCO^−^_3_) incorporation by tomato roots [33]. Furthermore, the *in vitro* rates of CA activity found in tomato roots were higher than the *in vitro* rates of PEPC activity [34] agreeing with the *in vivo* results in maize root tips [27].

In the present study, our histochemical and immunohistochemical data showed that CA activity and protein, as well as *GmCA1* gene expression pattern studied with *in situ* hybridization, coincide to tissues with the highest metabolic activity, such as the root apical meristem where cell division occurs, the middle layer of cortical parenchyma of root and hypocotyls where the cells are elongating and enlarging and the metaxylem and phloem vessels of root and hypocotyls which are still maturing. The co-localization of PEPC and CA suggests that a large fraction of the respired CO_2_ in the root and hypocotyls tissue may be re-introduced into C4 compounds. Zeeman and up Rees [35] reported a rate of carbon dioxide release of 0.028 μmol sucrose equivalents min^−1^·g^−11^ fresh weight from *Arabidopsis* roots. Furthermore, tobacco shows characteristics of C4 photosynthesis in cells of stem and petioles that surround the xylem and phloem while these cells are supplied with carbon for photosynthesis from the vascular system and not from stomata. These photosynthetic cells possess high activities of enzymes characteristic of C4 photosynthesis, which allow the decarboxylation of four-carbon organic acids from the xylem and phloem, thus releasing CO_2_ for photosynthesis [36]. Similarly, organic acids produced by PEPC activity in soybean roots and hypocotyls may translocate through the vascular system to other plant organs where they could be decarboxylated and the released CO_2_ may be reassimilated to form carbohydrates.

Our data indicated that high signal of CA activity and protein as well as *GmCA1* gene transcripts, were visible in the vascular tissue of roots and hypocotyls. A close examination of the expression patterns of different CAs in different cell types revealed that two gene loci of *Arabidopsis* encoding two distinct carbonic anhydrases are expressed at high levels in the stele [37]. In root steles, in which the vascular tissue is embedded in, low oxygen concentrations have been reported [38], while results from recent studies show that oxygen concentration is low inside the transport phloem *in planta* [39]. The meristematic and the root region of elongation have the greatest demand for O_2_ [40] so under low oxygen conditions, CA may facilitate oxygen diffusion inside the root, in ways similar to those suggested in legume nodules [41]. The joint action of CA and PEPC would increase malate production, resulting in increased cell osmotic potential, water influx, increased cell turgor and increased oxygen diffusion especially among cortex cells. Alternatively, CA may be involved in the control of bicarbonate/CO_2_ equilibrium across the different root cell layers playing a major role in the transport of O_2_ wherever there is gas-space continuum.

The relative activity of PEPC and PK has been compared in different growing tissues and seems to be highly variable. In sugar-fed maize root tips [42,43] and developing soybean seeds [44] as in tomato cells in exponential phase [45], three-fourths of triose-P provided by glycolysis have been found to be directed to tricarboxylic acid cycle through the PK. This slowing down of the PEPC flux could be compared with the interruption of this flux observed in corn root tips after 6h of carbon starvation [46]. This observation suggests that the PEPC activity was related to the supply of sugars to the cell. Roots of etiolated seedlings are actively growing and highly respiring heterotrophic tissues, in which a significant proportion of the carbon entering the glycolytic pathway is incorporated into numerous compounds such as amino acids, nucleic acids, fatty acids, and secondary metabolites [47]. Our temporal and spatial gene expression studies revealed that *GmPK3* and *GmPEPC7* are co-expressed in meristematic tissues of the root tip as well as in the central cylinder of etiolated soybean roots, suggesting that both PK and PEPC are involved in PEP metabolism in these tissues. Although accurate conclusions about which enzyme activity predominates can not be concluded from the present results, fine regulation of the PEP branch point may provide the plant with the necessary flexibility under a wide variety of physiological conditions.

## 4. Conclusions

In conclusion, our data showed that the pattern of cellular CA protein and activity localization as well as *GmCA1* spatial expression pattern in roots and hypocotyls of etiolated *G. max* seedlings essentially follows the spatial pattern of PEPC protein; suggesting that CA functions in accelerating carbon assimilation ensuring a continuous supply of bicarbonate to PEPC, which is able to reintroduce CO_2_ produced in respiration into metabolic intermediates. Furthermore, *GmPK3* expression, coding for a cytoplasmic isoform of PK, sharing the same substrate with PEPC, was detected in all different root cell types, indicating the necessary flexibility plant metabolism has to adopt in order to compensate various physiological conditions.

## 5. Experimental Section

### 5.1. Plant material and growth conditions

Soybean (*Glycine max* cv. Williams) seeds were pre-germinated on two sheets of moist paper, in Petri dishes, in darkness, at 26 °C, for 8 days. RNA was isolated from hypocotyls (HYP), and different parts of roots, representing the region of cell division (RT), the root region of elongation (MRI) and the root region of laterals emergence (MRII). The different parts of the root were determined using a stereoscope. For *in situ* hybridization, histochemical detection of enzyme activity and immune-histochemical detection of proteins, the roots from the 8-day old seedlings were used.

### 5.2. Characterization of a cDNA clone coding for GmPK3

By performing BLAST searches [48] among the soybean EST databases, many cDNA clones coding for a cytoplasmic isoform of PK were identified, obtained from ResGen, Invitrogen Corp., and their nucleotide sequences were determined. Alignment of these sequences allowed the identification of a distinct cDNA clone encoding a cytoplasmic isoenzyme of PK. As a part of its 5′ end was missing, total RNA was extracted from the roots of 8-day old seedlings [49] and a 5′ RACE-PCR was performed with the SMART 5′ RACE kit from Clontech (Westburg, NL). The amplified fragment was cloned into the pBlueScriptKS+ plasmid vector (Stratagene) and fully sequenced. The full-length cDNA clone was designated as *GmPK3* (NCBI accession number: CAI53675). The Clustal procedure was used for both analysis of the sequence data and determination of the relatedness of the deduced polypeptide to related sequences from other species.

### 5.3. RT-PCR analysis of GmPK3 and GmPEPC7 transcript levels

Semi-quantitative RT-PCR analysis was performed using total RNA extracted from the tissues of interest as described above and quantified by spectrophotometry and agarose gel electrophoresis. All RNA samples were treated with DNase I (Promega) according to the manufacturer’s instructions, to eliminate DNA contamination. In each case, the RT-PCR reactions were performed, according to the manufacturer’s directions (Qiagen) using gene-specific oligonucleotides as primers (GmPK3F: 5′-GGAGCTACTGATGTTGCC-3′, GmPK3R: 5′-CATGAGAAGCTCTAGCAG-3′, GmPEPC7F: 5′-ACCAGTTTGGATGCTGGG-3′, GmPEPC7R: 5′-ATCTGTACGGAAGGCAGC-3′). *GmPEPC7* cDNA has been isolated and characterized previously [16]. RNase inhibitor was obtained from Promega. For normalization of the different RNA preparations, a fragment of GmUBQ was amplified, using two gene-specific oligonucleotides as primers (GmUBQF: 5′-GGGTTTTAAGCTCGTTGT-3′, GmUBQR: 5′-GGACACATTGAGTTCAAC-3′). The reverse transcriptase reactions were performed in a thermal cycler at 50 °C for 30 min, followed by PCR amplification of 25-30 cycles at 94 °C for 1 min, 54 °C for 1 min and 72 °C for 1 min. A complete final extension for the PCR products was performed at 72 °C for 10 min. Amplified products were separated on 1.5% agarose gels, blotted on to nylon membranes and hybridized with the respective digoxigenin-11-rUTP labeled cDNA probes.

### 5.4. In situ hybridization

*In situ* hybridization of root segments was carried out essentially as described [50]. Root segments (5 mm) were fixed in 4% (w/v) paraformaldehyde supplemented with 0.25% (v/v) glutaraldehyde in 10mM sodium phosphate buffer (pH 7.4), for 1h, in a vacuum aspirator, dehydrated with ethanol, and then exchanged with xylene before embedding in paraffin. Sections (8 μm) were mounted on poly-l-lysine slides, digested with proteinase K for 30min at 37°C, treated with acetic anhydride, dried in ethanol, and then hybridized with appropriate gene specific digoxigenin-labeled probes overnight, at 42 °C. After washing with 4xSSC containing 5mM dithiothreitol, the slides were treated with RNase A for 30 min, at 37 °C, washed again at 37 °C with RNase A buffer (500 mM NaCl, 1 mM EDTA, 10 mM Tris-HCl, pH 7.5) containing 5mM dithiothreitol, and then processed for developing the digoxigenin antigen. This involved blocking with blocking reagent (0.5% blocking reagent in 100mM Tris-HCl, 150 mM NaCl, pH 7.5) for 30 min and BSA (1%BSA in 100 mM Tris-HCl, 150 mM NaCl, pH 7.5 containing 0.5% blocking reagent), followed by incubation with an anti-digoxigenin antibody conjugated to alkaline phosphatase (dilution 1:500 in 100 mM Tris-HCl, 150 mM NaCl, pH 7.5) for 2 h, and washing with blocking reagent (1%BSA in 100 mM Tris-HCl, 150 mM NaCl, pH 7.5 containing 0.5% blocking reagent) for 1h. The color was revealed by incubation in NBT and BCIP as long as necessary and the reactions were stopped with water, slides dehydrated, air-dried, and then mounted in DPX before viewing. Antisense and sense probes labeled with digoxigenin-11-rUTP (Roche) were used in parallel hybridizations. The sections were examined using a ZEISS Axiolab (Carl Zeiss, Jena, Germany) microscope and pictures were taken with SONY DSC-S75 38P/45 (SONY Corporation, Japan) digital camera system.

### 5.5. Histochemical localization of CA activity

CA activity was histochemically localized in *G. max* tissues as previously described [16]. Approximately 1–5 mm free-hand sections of *G. max* fresh roots and hypocotyls were cutted and fixed in 2% (w/v) paraformaldehyde, 2% (w/v) polyvinilopyrolidone (PVP) and 1 mM dithiothreitol (DTT) for 30 min at room temperature. The sections were washed in distilled H_2_O overnight at 4 °C and then incubated in the reaction mixture for 30 min. The reaction mixture was prepared fresh immediately before use by quickly mixing 17 mL of solution A containing 12 mM NiSO_4_, 180 mM H_2_SO_4_ and 39 mM KH_2_PO_4_ with 40 mL of solution B containing 220 mM NaHCO_3_. After incubation the sections were washed for 10 min in 0.67 mM phosphate buffer (pH 5.9). The developing of the histochemical color was performed by transferring the sections in 0.5% (v/v) (NH_4_)_2_SO_4_.

### 5.6. Total CA activity of G. max organ extracts

Freshly harvested tissues were homogenized in liquid nitrogen. Total CA activity was determined spectrophotometrically using a modification of the pH-indicator method [51]. Tissue extracts were mixed with 200 μL of reaction buffer [190 μL of 0.1M phosphate buffer pH: 6.5 and 10 μL of 0.1% (v/v) bromothymol blue] and 700 μL of 0.1M NaHCO_3_ and the increase in absorbance at 615 nm was monitored at 4 °C using a HITACHI U-2800 spectrophotometer. The observed rate was converted to equivalent μmol H^+^ consumed by comparison with a calibration established by titrating the reaction buffer with NaOH. The protein concentration was determined by the procedure of Bradford [52]. Data were analysed by one-way ANOVA.

### 5.7. Protein extraction from G. max tissues and Western blot of PEPC and CA

Approximately 0.5 g of soybean roots or hypocotyls were grounded in a mortar containing liquid nitrogen and the powder was mixed with equal volume of extraction buffer (125 mM Tris-HCl pH 6.8, 20% glycerol, 6% SDS) and centrifuged. Approximately 20 μg of total proteins were separated by sodium dodecyl sulfate-polyacrylamide gel electrophoresis (SDS-PAGE; 15% polyacrylamide) and then electrotransfered to nitrocellulose (Hybond-C extra; Amersham). Nonspecific binding sites on the membranes were blocked by 30 min incubation in TBST (20 mm Tris-HCl, pH 8.0, containing 150 mm NaCl, 0.05% Tween 20 and 1% BSA). The blots were probed with the antiserum raised against *Gm*PEPC7 and *Gm*CA1 [16] at a 1/2.500 dilution, in the blocking buffer, at room temperature, for 30 min. Blots were washed three times in TBST and then incubated with secondary antibody (alkaline phosphatase conjugated anti-rabbit IgG at dilution 1:1000 in TBST). Blots were again washed three times in TBST and the color was revealed by incubation in NBT and BCIP as long as necessary and the reactions were stopped with water.

### 5.8. Immunohistochemical detection of PEPC and CA

Approximately 5 mm root segments were embedded in paraffin and sectioned as described for the *in situ* hybridization method. 8 μm sections were blocked as previously reported and incubated overnight with antiserum raised against *Gm*PEPC7 and *Gm*CA1 [16]. Briefly, sections were incubated in 3% BSA in TBST (20 mM Tris-HCl, 150 mM NaCl, 0.05% Tween20, pH 7.5) for 1h and then in the antibody solutions (pre-immune serum and immune serum were used at dilution of 1:1500 in TBST) for 14 h. Sections were washed with TBST for 15 min and then incubated with secondary antibody (alkaline phosphatase conjugated anti-rabbit IgG at dilution 1:500 in 1% BSA in TBST). Sections incubated with pre-immune serum were used as control. The color was revealed by incubation in NBT and BCIP as long as necessary and the reactions were stopped with water. The sections were examined using a ZEISS Axiolab (Carl Zeiss, Jena, Germany) microscope and pictures were taken with SONY DSC-S75 38P/45 (SONY Corporation, Japan) digital camera system.

## Figures and Tables

**Table 1.** Carbonic anhydrase activity for plumule, hypocotyls and roots of 8-day old etiolated soybean seedlings.

**CA activitiy [μmole H^+^min^−1^ (mg protein)^−1^]**		
**plumule**	**hypocotyl**	**root**
ND (a)	85.527±8.785 (b)	216.363±51.969 (c)

Means±STADEV are shown (n=3).

^(a)^ ND: not detected.

^(b,c)^ Values with different letters are significantly different at p<0.05 (one-way ANOVA).
